# E2F2 promotes lung adenocarcinoma progression through B-Myb- and FOXM1-facilitated core transcription regulatory circuitry

**DOI:** 10.7150/ijbs.72386

**Published:** 2022-06-25

**Authors:** Kailong Du, Shijie Sun, Tinghui Jiang, Tao Liu, Xiaofeng Zuo, Xing Xia, Xianjun Liu, Yitao Wang, Youquan Bu

**Affiliations:** 1 Department of Biochemistry and Molecular Biology, College of Basic Medical Sciences, Chongqing Medical University, Chongqing 400016, China.; 2 Molecular Medicine and Cancer Research Center, Chongqing Medical University, Chongqing 400016, China.

**Keywords:** Lung adenocarcinoma, E2F2, Transcriptional regulation, B-Myb, FOXM1

## Abstract

Lung adenocarcinoma (LUAD) causes severe cancer death worldwide. E2F2 is a canonical transcription factor implicated in transcription regulation, cell cycle and tumorigenesis. The role of E2F2 as well as its transcription regulatory network in LUAD remains obscure. In this study, we constructed a weighted gene co-expression network and identified several key modules and networks overrepresented in LUAD, including the E2F2-centered transcription regulatory network. Function analysis revealed that E2F2 overexpression accelerated cell growth, cell cycle progression and cell motility in LUAD cells whereas E2F2 knockdown inhibited these malignant phenotypes. Mechanistic investigations uncovered various E2F2-regulated downstream genes and oncogenic signaling pathways. Notably, three core transcription factors of E2F2, B-Myb and FOXM1 from the LUAD transcription regulatory network exhibited positive expression correlation, associated with each other, mutually transactivated each other, and regulated similar downstream gene cascades, hence constituting a consolidated core transcription regulatory circuitry. Moreover, E2F2 could promote and was essentially required for LUAD growth in orthotopic mouse models. Prognosis modeling revealed that a two-gene signature of E2F2 and PLK1 from the transcription regulatory circuitry remarkably stratified patients into low- and high-risk groups. Collectively, our results clarified the critical roles of E2F2 and the exquisite core transcription regulatory circuitry of E2F2/B-Myb/FOXM1 in LUAD progression.

## Introduction

Lung cancer accounts for the highest cancer incidence and mortality around the world 1. Lung adenocarcinoma (LUAD), one of the prominent histological subtypes of lung cancer, accounts for about 40% of lung malignancies 2. Development of LUAD is related to smoking, carcinogens and air pollution 3. Accumulated genomic studies have found a series of driver genes for LUAD, such as TP53, EGFR, ALK, ROS1, KRAS, KEAP1, STK11, and NF1 4, 5. LUAD is predominantly asymptomatic at its early stages and is often diagnosed at an advanced stage for lack of effective diagnostic screening 6. Targeted therapies against several LUAD driver genes increase the 5-year survival rate of advanced LUAD by four to five times in recent years 7, 8. However, acquired resistance often develops after a period time of treatment and there is still a large percent of LUAD without treatment target. Thus, more efforts are urgently needed to functionally identify more LUAD-associated genes or key molecular networks to develop novel early diagnostic and therapeutic strategies for the treatment of LUAD.

The E2F (the adenoviral early region 2 binding factor) family of transcription factors consists of eight members (E2F1-E2F8), and is classified into activators (E2F1-E2F3), repressors (E2F4-E2F6), and atypical repressors (E2F7, E2F8) on the basis of transcriptional function 9-11. All the eight E2F family members are critical regulators in cell cycle and cell proliferation, and also participate in differentiation, apoptosis and other physiological and pathological processes 11-16. However, the specific and overlapping functions of E2F1-E2F8 at the biochemical level have not been fully elucidated 11. As for E2F2, in addition to its putative canonical role in cell cycle and cell proliferation, its role in tumorigenesis has been also documented by several groups in several types of cancers, including ovarian cancer, breast cancer, colorectal cancer, etc 17-21. Our group has recently reported that E2F2 collaborates with another canonical transcription factor B-Myb to accelerate colorectal cancer progression through a reciprocal feed-forward transactivation loop 21. However, the role of E2F2 as well as the E2F2-regulated transcription network in LUAD remains elusive.

In this study, we first systematically analyzed and identified the critical dysregulated modules and networks as well as the hub genes at the whole genomic level in LUAD, and found that E2F2 is a critical transcription factor that plays a tumor-promoting role in LUAD. We further demonstrated that E2F2 collaborates with B-Myb and FOXM1 to constitute an exquisite core transcription regulatory circuitry that contributes to human LUAD progression.

## Materials and Methods

### TCGA Data Acquisition and Preprocessing

LUAD transcriptome data and clinical data were downloaded from The Cancer Genome Atlas (TCGA) data portal 22. From a total of 592 LUAD samples, 55 pairs of adjacent non-tumor tissue samples and tumor tissue samples were selected for gene expression network analysis. The clinic characteristics of the patients are shown in Table S1. To facilitate the follow-up analysis, the gene expression matrix was filtered based on the following criteria: the genes that show null expression values in more than 30 samples are removed. The genes without corresponding annotation information were also removed. To ensure that the results of network construction are reliable, outlier samples were removed by sample cluster analysis.

### Construction of Weighted Gene Co-Expression Network

Construction of co-expression networks and identification of co-expression modules were conducted by using the WGCNA R package in R 3.6.2 23. The similarity of gene expression profiles was evaluated by calculating the Pearson correlation coefficient between all gene pairs, and a power (beta) was defined according to the scale-free standard, which converted the node-degree distribution in the similarity matrix into log-linear, thus generating an appropriate adjacency matrix (aij). The topological overlap matrix (TOM) is computed:

TOMij = (lij + aij) / [min (ki, kj) + 1 - aij] (1)

where lij is defined as the dot product on row i and column j in adjacency matrix, and ki (the connectivity) is the summation of row i in adjacency matrix. 1-Tom was hierarchically clustered by using the “average” clustering method, and the module was identified by using the dynamic tree cutting method.

### Network Visualization

The genes in the turquoise module were screened based on the weight value (the adjacency threshold value containing edge in the output) greater than 0.2. The LUAD module total network was constructed in Cytoscape3.6.1 software 24. Then, the network was analyzed by molecular complex detection (MCODE) 25. MCODE is a Cytoscape plug-in, which can define the densely connected areas in the network that may represent molecular complexes as subnetworks. In the network, molecules with the same complex structure often show a high degree of correlation, which can be detected by plug-in, and cutoff criteria were set as node score cutoff at 0.2, K-Core at 2, degree cutoff at 2 and Max Depth at 100 to obtain important subnetworks. Then the differentially expressed genes obtained by analyzing TCGA LUAD dataset were intersected with the genes in the sub-network. Finally, the LUAD module key network was defined.

### Association of Modules with Clinical Traits

The clinical data of LUAD include sex, age, tumor pathologic stage, lymph node pathologic stage, metastasis stage, sample type, vital status, etc. The association between each module and each trait was first represented by the average gene expression level of the module gene members, and then the module average was associated with the observed phenotypic traits using Pearson correlation analysis. Modules with significant association to phenotype were obtained. Finally, in order to further confirm the modules that are significantly related to the phenotype, the labeled heatmap function was used to calculate the correlation coefficient between module membership (gene expression level) and gene significance (GS, used to assess the association between genes and phenotype).

### Functional Enrichment Analysis and Hub Gene definition

To better explore the biological significance of the LUAD module key network, the DAVID v6.8 (The Database for Annotation, Visualization and Integrated Discovery) 26 and ClueGO v2.5.5 27 were utilized to conduct gene ontology analysis and Reactome pathway analysis. ClueGO is a Cytoscape plug-in that extracts representative functional biological information for large lists of genes or proteins. In order to explore the potential transcription regulatory networks in LUAD, the LUAD module TF network was finally established by intersecting the LUAD module key network with the transcription factor list.

### qRT-PCR and Immunoblotting analysis

Total RNA was extracted from cells by using Trizol reagent (Invitrogen) and qRT-PCR was performed by using SYBR PremixEx Taq^TM^ (TaKaRa) as previously described 21, 28. For immunoblotting analysis, cells were lysed with RIPA buffer (Beyotime) supplemented with protease inhibitor cocktail (Bimake), then total protein lysates were centrifuged and boiled with loading buffer. All protein lysates were analyzed by SDS-PAGE. The information of the primers and antibodies was provided in Table S2 and S3.

### Lentivirus-mediated E2F2 overexpression and knockdown cell establishment

pCDH-puro-HA-E2F2 lentiviral expression vector was previously constructed 21. The short hairpin RNA (shRNA) targeting human E2F2 (E2F2 shRNA) and negative control shRNA (NCshRNA) oligonucleotides were Synthesized by Sangon (Shanghai, China), then annealed and cloned into pLKO.1-puro lentiviral vector. The oligonucleotide sequence was provided in Table S2. Recombinant lentiviral particles were prepared according to the previous protocol 21, 28, 29. Forty-eight hours after lentivirus infection, cells were selected in the presence of puromycin(1μg/ml) for 72h to generate the stable E2F2 overexpression or knockdown cells.

### Cell culture, siRNAs, and transfection

Human LUAD cell lines, A549 and H1975, were obtained from Chinese Academy of Sciences Shanghai cell bank (Shanghai, China). Human embryonic kidney cell line 293Ta was purchased from FulenGen (Guangzhou, China). Cells were routinely maintained in DMEM, MEM, or RPMI1940 medium (Hyclone) supplemented with 10% of fetal bovine serum (Hyclone), penicillin (10^7^ U/L) and streptomycin (10 mg/L) in a humidified incubator containing 5% CO2 at 37 °C. Cell lines were routinely tested for short tandem repeat authentication and mycoplasma contamination 21. The short interfering RNAs (siRNA) were chemically synthesized by GenePharma (Shanghai, China), and were transiently transfected into cells using Lipofectamine RNAiMAX reagent (Invitrogen). The sequences of the siRNAs were provided in Table S2. The overexpression plasmids were transiently transfected into cells using Lipofectamine 3000 reagent (Invitrogen).

### Cell cycle, cell growth, cell viability and cell motility assays

Cell cycle analysis was conducted as described previously 30. Cell growth and cell mobility were monitored by JULI Stage Real-time Cell History Recorder (NanoEntek, Seoul, South Korea) as described previously 30. Images were taken, and the growth rate and cell mobility ability were quantified. Cell viability was analyzed by trypan blue (Solarbio Life Sciences, Beijing, China) staining. Cell numbers were counted by CellDrop FL Fluorescence Cell Counter (Devovix, USA). Cell growth was also checked by CCK-8 Kit (Bimake, China) 21, 28. Wound-healing assays were conducted as described previously 28.

### RNA-seq analysis

Total RNA was extracted from the E2F2 knockdown cells and its corresponding negative control cells in exponentially growing state, and cDNA libraries were then constructed and subjected to RNA-seq analysis as described previously 29. Differentially expressed genes were subjected to GO and KEGG Pathway enrichment analysis. Gene Set Enrichment Analysis (GSEA) was carried out to identify the oncogenic signatures and pathways significantly altered 21, 31.

### EdU labelling and indirect immunofluorescence assays

EdU labelling assays were conducted by using Cell-Light EdU Apollo488 *In vitro* Kit (C10310-3, RiboBio, China) following the manufacturer's instructions. Briefly, the exponentially growing cells were seeded on 96-well plates and then sequentially labelled with 10 μM EdU for 2h, fixed, stained with Apollo488 and Hoechst 33342 (DNA dye for nuclei staining), and finally observed under fluorescence microscope (Leica, Germany).

The indirect immunofluorescence assays were conducted as described previously with minor modifications 21. Briefly, cells were sequentially subjected to fixation, permeabilization, blocking, and stepwise incubations with the primary antibody as well as the secondary antibody. Cell nuclei were stained with 4', 6-diamidino-2-phenylindole (DAPI, D9542, Sigma). Cells were observed under laser scanning confocal microscope. The antibodies used were listed in Table S3.

### Co-immunoprecipitation (Co-IP) assays

Co-IP assays were carried out as described previously 21. Briefly, H1299 cells were transiently co-transfected with the pCDH-puro-HA-E2F2, LV105-Flag-B-Myb and GV365-3×Flag-FOXM1b expression constructs. Whole cell extracts were prepared and precleared with Protein A/G Magnetic Beads for 1 h at 4 °C, and then incubated with primary anti-HA antibody (Sigma) and normal rabbit IgG (Beyotime) overnight at 4 °C. The antigen-antibody complexes were then captured by Protein A/G Magnetic Beads. Magbeaded immunoprecipitates were then separated by Magnetic separator, and finally subjected to immunoblotting analysis with the indicated antibodies.

### Protein-protein docking analysis

The 3D structures of E2F2 and FOXM1 were predicted and downloaded with high model confidence (≥70) from the AlphaFold Protein Structure Database 32. The crystal 3D structure of FOXM1 was also downloaded from RSCB PDB and HDOCK. Protein-protein docking analysis was carried out using the HDOCK server 33. Three top-scored models of potential interaction between FOXM1 and E2F2 were provided.

### Tumor xenografts

BALB/c-nude mice (female, 5-6 weeks of age, weighing 18-20 g) were randomly distributed into the indicated groups (n=5), and 2× 10^6^ cells were injected subcutaneously into the mice. Tumor volume was calculated using the equation L × W^2^/ 2 (L = length, W = width). After 32 days, the mice were sacrificed and the tumor tissues were harvested and frozen in liquid nitrogen or fixed in formalin and embedded in paraffin using the routine method. All procedures conformed to the legal mandates and guidelines of the Laboratory Animal Center of Chongqing Medical University for the care and maintenance of laboratory animals.

### Luciferase reporter assays

The promoter reporter E2F2-P1314(-984/+329) and B-Myb-P1064(-916/+148) were constructed previously 21. The promoter regions of PLK1 (-768/+74) were obtained by PCR amplification and cloned into pGL3-basic vector to generate PLK1-P842(-768/+74) reporter. For luciferase reporter assays, equal number of cells were seeded into 12-well plates in triplicate and co-transfected with the corresponding plasmids when the confluence of cells reaches 50-60%. Forty-eight hours after transfection, the luciferase activities were determined using the Dual-Luciferase assay system (Promega) as described previously 34. Firefly luciferase activities were mainly used to indicate the promoter activities.

### Chromatin Immunoprecipitation (ChIP) assays

ChIP assays were carried out using the EZ ChIP™ Chromatin Immunoprecipitation kit (Upstate, Lake Placid, NY, USA) as described in the previous study 34. The sequences of the primers are provided in Table S2, and the information of antibodies was offered in Table S3.

### Construction and assessment of prognostic risk score model for LUAD

Based on the cut-off value of the median survival time, LUAD patients were divided into high-risk (n=252) and low-risk (n=252) groups, and Kaplan-Meier survival curve was generated to predict cases with low or high risk. Univariate Cox regression was used to estimate the association between the expression level of each gene of the LUAD module TF network and patient's overall survival (OS). Multivariate Cox regression with stepwise regression method was conducted to screen and eliminate the variables causing multicollinearity within the genes of LUAD module TF network. The formula based on gene expression level to calculate the risk score for predicting prognosis was finally established as following:

RS = ExpmRNA1× βmRNA1+ ExpmRNA2×βmRNA2+ … + ExpmRNAn× βmRNA (2)

ExpmRNA represents the expression level of each gene, and βmRNA denotes the regression coefficient of the gene in the multifactor cox regression model 35, 36.

### Statistical analysis and routine bioinformatic analysis

Routine statistical analyses were conducted using SPSS (Statistical Product and Service Solutions) version 21 software package (SPSS Inc, Chicago, USA). The difference between different groups was quantitatively estimated by t-test. Statistical tests with P values less than 0.05 were considered significant. As for routine bioinformatic analysis, R software (version 3.6.2 and 4.1.0), the DESeq2 package, the survival package and the corrr package were utilized to identify the differentially expressed genes, survival estimation and gene expression correlation analysis, respectively. The pheatmap package of R3.6.2 was used to construct heat maps that visualize the differential gene expression.

## Results

### Construction of Weighted Gene Co-Expression Network for LUAD

**Figure 1:**
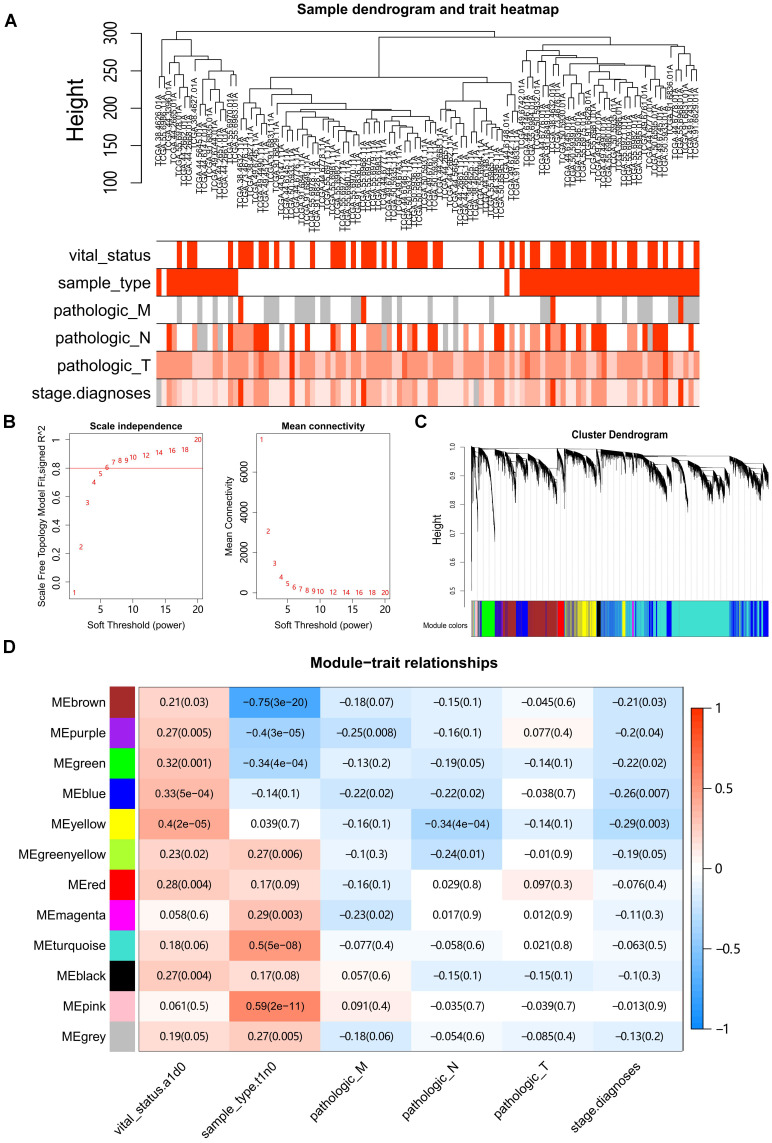
** Construction of Weighted Gene Co-Expression Network.** (**A**) Clustering of samples with removed outliers. White-to-red linear gradient colors were associated with the indicated clinical variables, and grey for missing data. (**B**) Analysis of network topology for soft-thresholding powers. (**C**) Hierarchical clustering dendrogram of identified co-expressed genes in modules, each colored row denotes a color-coded module that contains a group of highly connected genes. (**D**) Heatmap of the correlation between modules and clinical features. Each row corresponds to a module, each column corresponds to a clinical feature, and each cell contains a corresponding Pearson correlation coefficient and P value.

Hierarchical clustering of the LUAD samples was conducted based on Euclidean distance computed on gene expression data, and integrated with the clinical information of patients (Fig. 1A). Four outlier samples were removed (Fig. S1A). Network topology analysis ensured a scale-free topology network with the defined soft-thresholding power of six (Fig. 1B). A total of 12 modules were identified based on the dynamic tree cutting algorithm with the parameters of minModuleSize at 30 and mergeCutHeight at 0.25, (Fig. 1C). For each module, the eigengene (the first component expression of genes in module) was determined, and the correlations of eigengenes with clinical traits such as tumor stage and grade were then subsequently calculated. The results showed that four modules were mostly associated with one or more traits (Pearson's correlation coefficient >0.35, Fig. 1D). The turquoise, pink, brown and purple modules were significantly correlated with the sample type (tumor vs normal), whereas the yellow module showed the highest correlation with survival status in LUAD.

### Identification of critical modules and networks in LUAD

**Figure 2:**
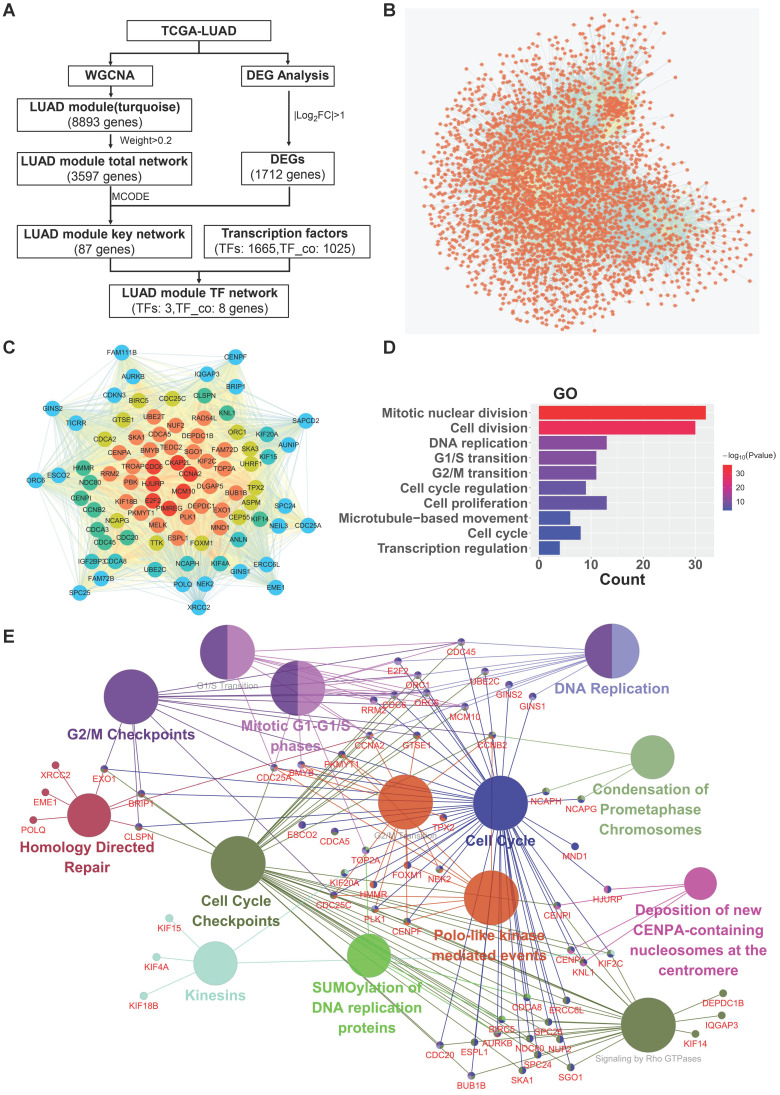
** Critical modules and networks in LUAD.** (**A**) Network visualization flowchart. (**B**) The LUAD module total network. Nodes represent genes, edges represent two genes are related. (**C**) The LUAD module key network. From the edge of the network to the middle, the color gradually changes, and the genes closer to the middle have more edges. (**D**) Biological process (GO) enrichment analysis result of the LUAD module key network. (**E**) Reactome Pathway enrichment analysis result of the LUAD module key network.

To screen and identify potential hub oncogenic driver genes in LUAD, we selected the turquoise module, which shows significant positive correlation with the phenotype of tumor vs normal and contains the largest number of genes, for further deep network analysis in combination with LUAD differential expressed genes (DEGs) analysis (Fig. 2A for workflow in details). The turquoise module contained 8,893 genes, showing significant correlation between turquoise module membership and gene significance (Fig. S1B). The LUAD turquoise module genes with “weight” value ≥ 0.2 were then used to construct the “LUAD module total network” (Fig. 2B). The sub-network with the highest MCODE score was further identified using the plug-in Molecular Complex Detection (MCODE) in Cytoscape. The LUAD differentially expressed genes (DEGs) with |log2FC| ≥ 1.0 were then identified and intersected with the genes in the sub-network, and subsequently used to establish the “LUAD module key network” (Fig. 2C). The GO analysis showed that the “LUAD module key network” genes were mainly enriched in the biological processes (BP) such as mitotic nuclear division, cell division, DNA replication, cell proliferation, regulation of transcription in cell cycle, etc. (Fig. 2D). Likewise, the pathway enrichment analysis also demonstrated that the “LUAD module key network” genes were mainly implicated in pathways such as cell cycle, DNA replication, G2/M transition, G1/S transition, etc. (Fig. 2E).

### E2F2-centered transcription regulatory network is overexpressed in LUAD

**Figure 3:**
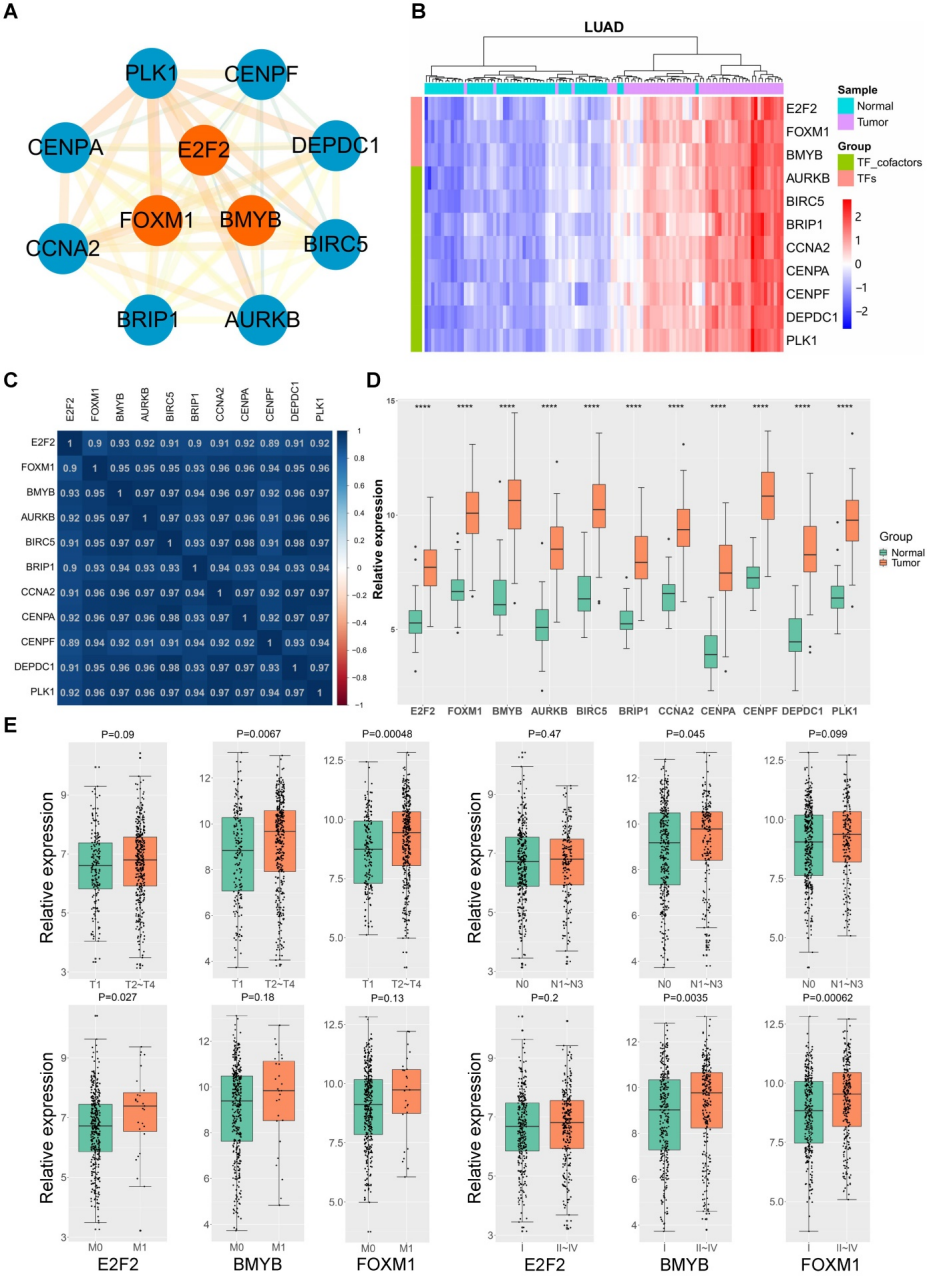
** LUAD TF network and hub genes.** (**A**) The LUAD module TF network. Orange nodes represent transcription factors, blue nodes represent co-transcription factors. (**B**) Heatmap of the expression levels of LUAD module TF network genes in LUAD tissues in comparison with normal counterparts. (**C**) Heatmap of Pearson correlation coefficient between LUAD module TF network genes in LUAD. (**D**) The LUAD module TF network genes are upregulated in LUAD. P<0.0001 (****). (**E**) The expressions of E2F2, B-Myb and FOXM1 are correlated with TNM status and clinical stages of LUAD. The gene expression levels in (D) and (E) are estimated and expressed as log_2_(count+1), in which “count” stands for read counts of each gene from RNA-seq data.

To gain an insight into the driving force in the “LUAD module key network”, a total of 11 genes encoding three human transcription factors and cofactors were identified and used to finally construct the “LUAD module TF network” (Fig. 3A). Among the 11 transcription regulators, three of them were transcription factors (E2F2, B-Myb, FOXM1) and seven are transcription co-factors (AURKB, BIRC5, BRIP1, CCNA2, CENPA, CENPF, DEPDC1, and PLK1). Notably, the expression of these 11 genes were significantly correlated with each other in LUAD, and remarkably upregulated in tumor tissues compared with the normal counterparts (Fig. 3B-D). In addition, detailed analysis showed that the expression of some of these genes were also correlated with LUAD stage and grade (Fig. 3E). As transcription factors play a central role in maintaining cell phenotypes, we then focused on the E2F2, B-Myb and FOXM1. Given that the functional implications of B-Myb and FOXM1 in LUAD have been established, E2F2 was then chosen to be subject to detailed functional analysis in LUAD.

### E2F2 promotes LUAD cell growth

**Figure 4:**
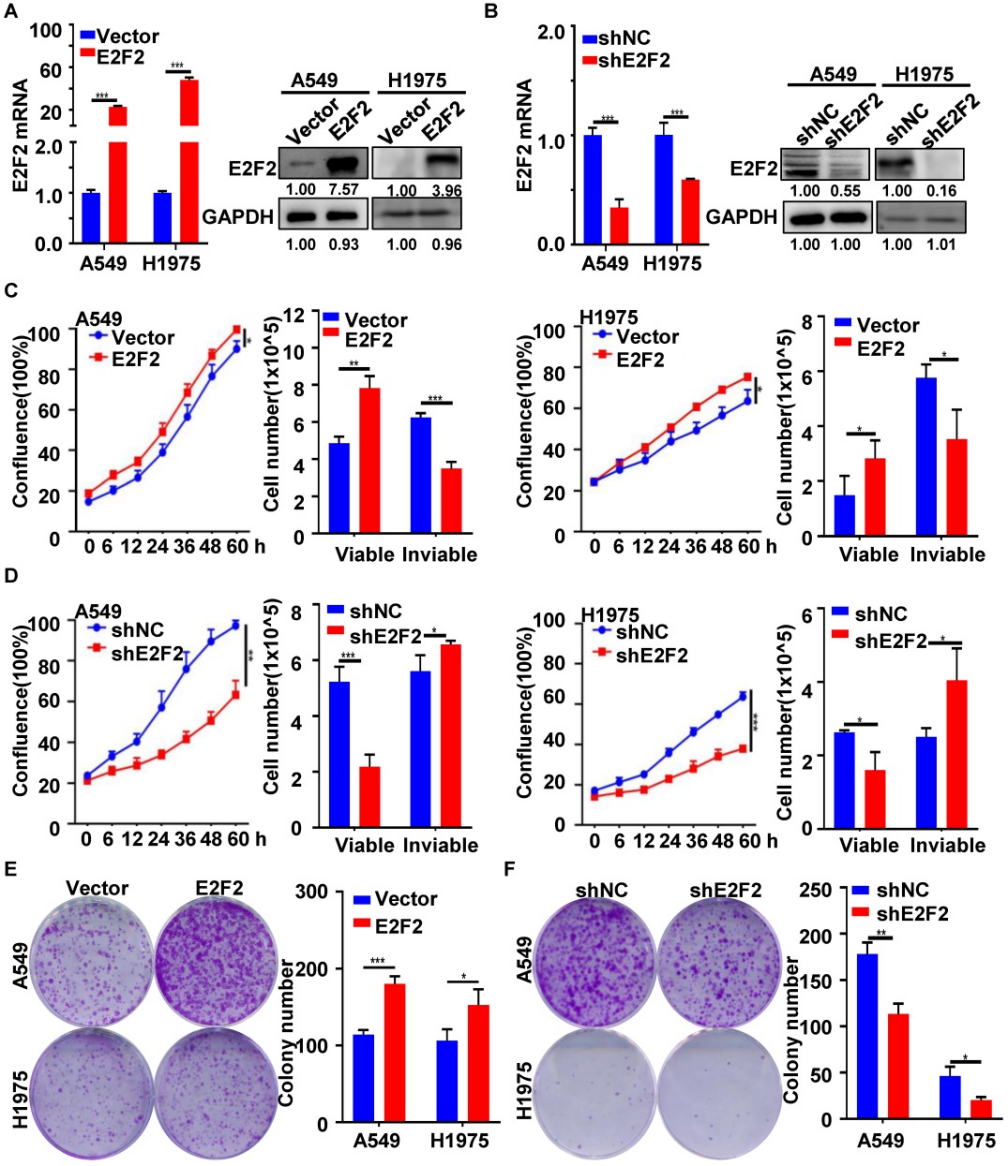
** E2F2 accelerates LUAD cell growth.** (**A**) Lentivirus-mediated stable E2F2 overexpression. The empty control and E2F2-expressing lentivirus particles were used to infect A549 and H1975 cells, and selected with puromycin to obtain the control (LV-vector) and E2F2 (LV-E2F2) overexpressing stable cells. E2F2 expression was determined by qRT-PCR and immunoblotting. (**B**) Lentivirus-mediated stable E2F2 knockdown. Stable cells were generated by the lentivirus particles expressing negative control shRNA (shNC) and E2F2 shRNA (shE2F2). (**C-D**) E2F2 promotes cell proliferation. Cell growth was monitored by JULI Stage Real-time Cell History Recorder, and cell viability was determined by Trypan blue exclusion assays at the end of time-points. (**E-F**) E2F2 enhances colony formation. Cells were seeded on 6-well plates to detect the anchorage-dependent colony formation ability. Each bar represents the mean ± SD value from at least three independent experiments. *P < 0.05, **p < 0.01, ***p < 0.001.

To better understand the function of E2F2 in lung adenocarcinoma progress, two different LUAD cell lines (A549, H1975) were used to generate stable E2F2 overexpression or knockdown cells. E2F2 overexpression and knockdown effects were verified by qRT-PCR and immunoblotting analyses (Fig. 4A and 4B). Real-time cell growth monitoring, Trypan blue exclusion and CCK8 assays demonstrated that E2F2 overexpression enhanced the growth rate and viability of both cell lines, whereas knockdown E2F2 inhibited that (Fig. 4C and 4D, Fig.S2). Anchorage-independent colony formation assays indicated that overexpression of E2F2 could increase the capacity of colony formation in both A549 and H1975 cells, whereas knockdown of E2F2 significantly decreased the number of colonies (Fig. 4E and 4F). Taken together, these findings suggest that E2F2 promotes LUAD cells growth *in vitro*.

### E2F2 accelerates LUAD cell cycle progression

**Figure 5:**
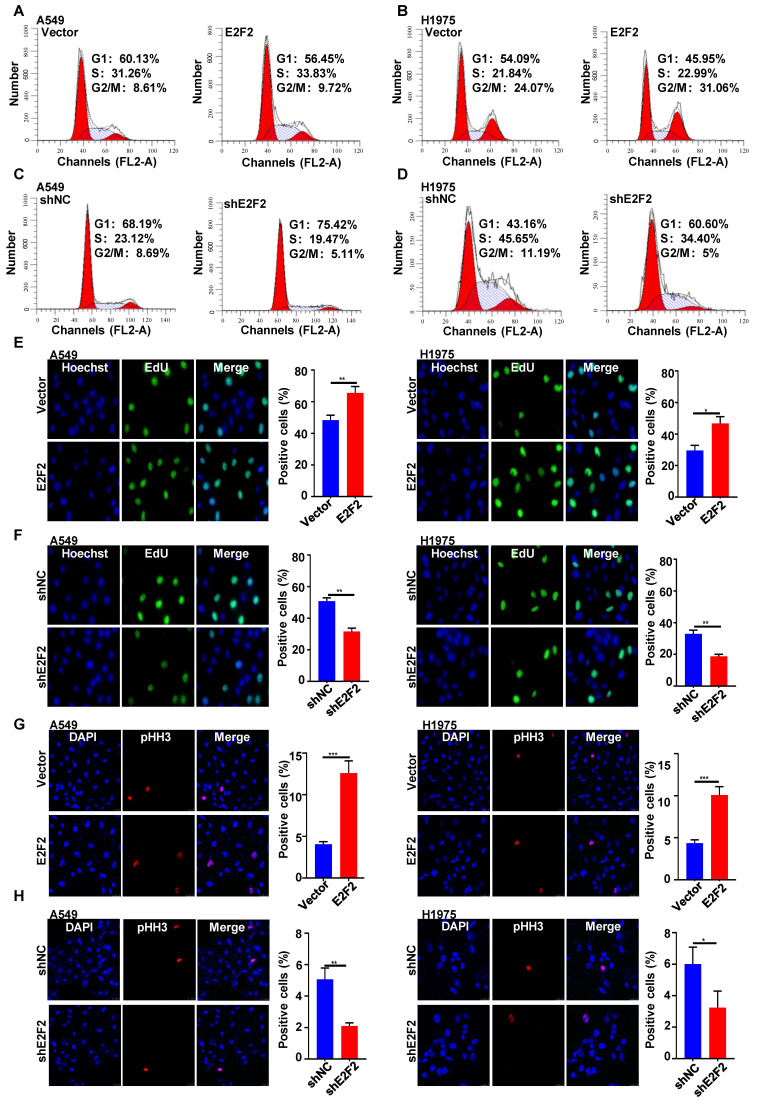
** E2F2 promotes LUAD cell cycle progression.** (**A-D**) Cell cycle distribution. Cells were seeded on six-well plates, and then collected for flow cytometer analysis at an exponentially growing state. The experiment has been repeated at least three times, and a representative cell cycle phase histogram was provided for each assay. (**E-F**) EdU labeling. DNA biosythensis was labeled by EdU (green), and cell nuclei were stained by DAPI (blue) in the exponentially growing E2F2 knockdown and overexpression cells. The EdU positive cells were calculated for statistical analysis. (**G-H**) Phospho-histone H3 (pHH3) staining. The stable E2F2 overexpression and knockdown cells were stained with anti-phospho-histone H3 (pHH3) antibody (red). The pHH3 positive cells were counted for statistical analysis.

Next, we investigated the functional involvement of E2F2 in cell cycle in LUAD cells. Flow cytometry analysis showed that E2F2 overexpression resulted in progression into S and G2/M phases, as indicated by the increased percentages of S and G2/M phase cells (Fig. 5A and 5B). Conversely, E2F2 knockdown caused significant delayed entry into S and G2/M phases with accumulated cells in G1 phase in both A549 and H1975 cells (Fig. 5C and 5D). Accordingly, EdU labeling analysis revealed that E2F2 overexpression promoted DNA biosynthesis while E2F2 knockdown inhibited that (Fig. 5E and 5F). In addition, phospho-histone H3 (pHH3) staining further validated that E2F2 overexpression increased the number of phospho-histone H3-positive mitotic cells when compared with the control group cells, while E2F2 knockdown had the opposite effect (Fig. 5G and 5H). These results indicate that E2F2 promotes cell cycle progression of LUAD cells.

### E2F2 enhances LUAD cell motility

**Figure 6:**
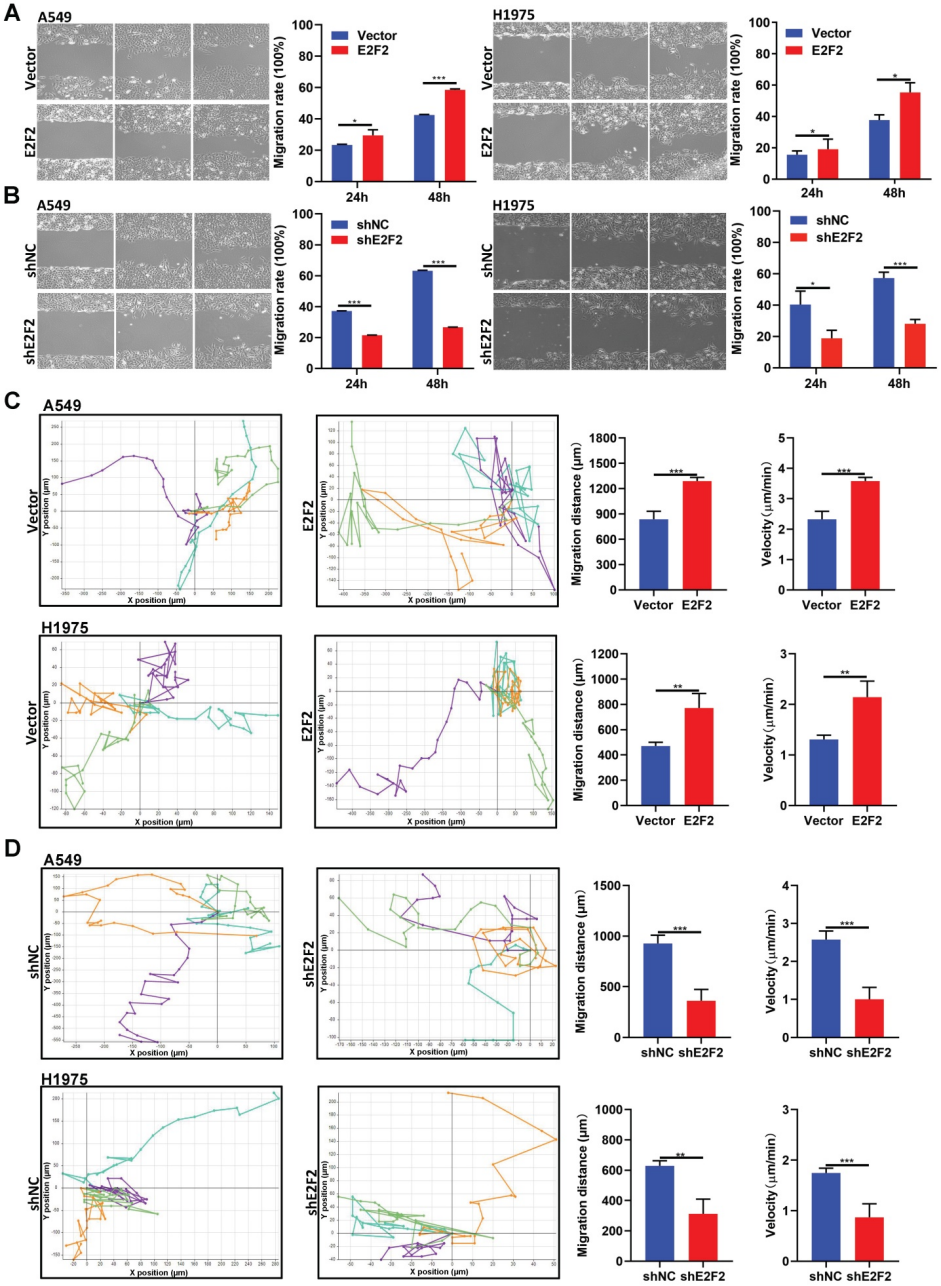
** E2F2 enhances LUAD cell motility.** (**A-B**) Wound healing assays. The scratches were introduced in the stable E2F2 overexpression and knockdown cells, and then continuously observed for cell migration abilities. (**C-D**) Cell motility assays. The motilities of the stable E2F2 overexpression and knockdown cells were monitored using JULI Stage Real-time Cell History Recorder, and the motile trajectories of selected cells as well as the calculated mean minutely migration speeds were presented.

We then investigated whether E2F2 affects LUAD cell motility. The wound healing assays demonstrated that E2F2 overexpression increased the rate of wound closure both in A549 and H1975 cells (Fig. 6A). Conversely, the migratory abilities of A549 and H1975 cells were decreased by E2F2 knockdown (Fig. 6B). In accordance with these findings, cell motility assays by a live cell imaging system showed that E2F2 overexpression accelerated the total migration distance and average velocity of A549 and H1975 cells compared with control groups (Fig. 6C), whereas E2F2 knockdown had the opposite effect on both cells (Fig. 6D). These results strongly indicate that E2F2 could increase LUAD cell migration and motility.

### Identification of downstream target genes and pathways regulated by E2F2

**Figure 7:**
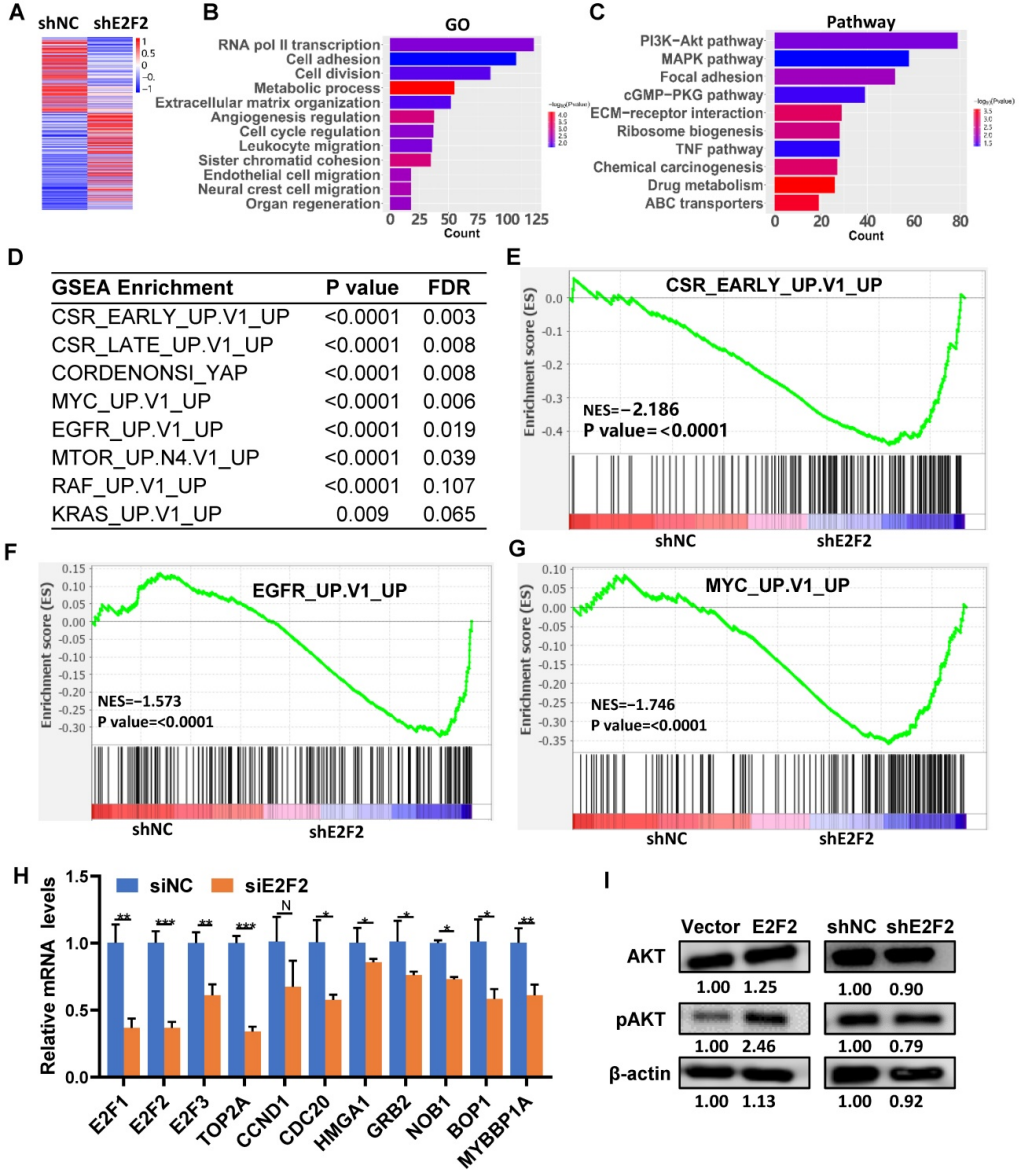
** E2F2 regulates key signaling pathways and oncogenic signatures.** (**A**) Heatmap of the differentially expressed genes regulated by E2F2. The stable E2F2 knockdown A549 cells were collected and subjected to RNA-seq analysis. (**B-C**) GO and KEGG pathway enrichment analysis of the differentially expressed genes. (**D**) Top enriched gene sets by GSEA analysis. False discovery rate, FDR. (**E-G**) GSEA plots of the top enriched gene sets. (**H**) Verification of important E2F2-regulated downstream genes by qRT-PCR in E2F2 knockdown cells. (**I**) E2F2 is essential for activation of PI3K-AKT pathway. Immunoblotting was conducted to determine total and phosphorylated AKT (pAKT) levels in the stable E2F2 overexpression and knockdown A549 cells.

To explore the intrinsic molecular mechanism of E2F2 promoted malignant phenotypes in LUAD cells, RNA-seq analysis was used to determine the transcriptomic changes in E2F2 knockdown cells. A total of 4492 differentially expressed genes (DEGs) were found after E2F2 knockdown (1955 genes down-regulated and 2537 genes up-regulated) (Fig. 7A). GO analysis demonstrated that the DEGs were enriched in transcription regulation, cell cycle regulation, cell proliferation, cell motility, cell migration, etc. (Fig. 7B). Pathway analysis indicated that the DEGs were involved in multiple tumor-associated pathways, such as PI3K-Akt signaling, MAPK signaling, eGMP-PKG signaling, TNF signaling pathways, etc. (Fig. 7C, Table S4). Furthermore, using the GSEA-4.0.3V for gene set enrichment analysis (GSEA), a number of cancer-related gene sets were enriched, including serum-stimulated genes (CSR_LATE_UP.V1_UP), MYC activated genes (MYC_UP.V1_UP), EGFR activated genes (EGFR_UP.V1_UP), etc. (Fig. 7D-G). GSEA analysis also revealed similarly enriched GOs and Pathways (Fig. S3, Table S5). A series of key downstream genes of E2F2 were confirmed by qRT-PCR, such as E2F1, TOP2A, CDC20, HMGA1, GRB2, NOB1 (Fig. 7H). In addition, overexpression of E2F2 could increase phosphorylation of AKT in A549 cells, while E2F2 knockdown showed the opposite effect (Fig. 7I). These results indicate that E2F2 may be an important regulator of multiple cancer-related signaling pathways in LUAD cells.

### Interconnected coregulatory circuitry among E2F2, B-Myb and FOXM1 in LUAD

**Figure 8:**
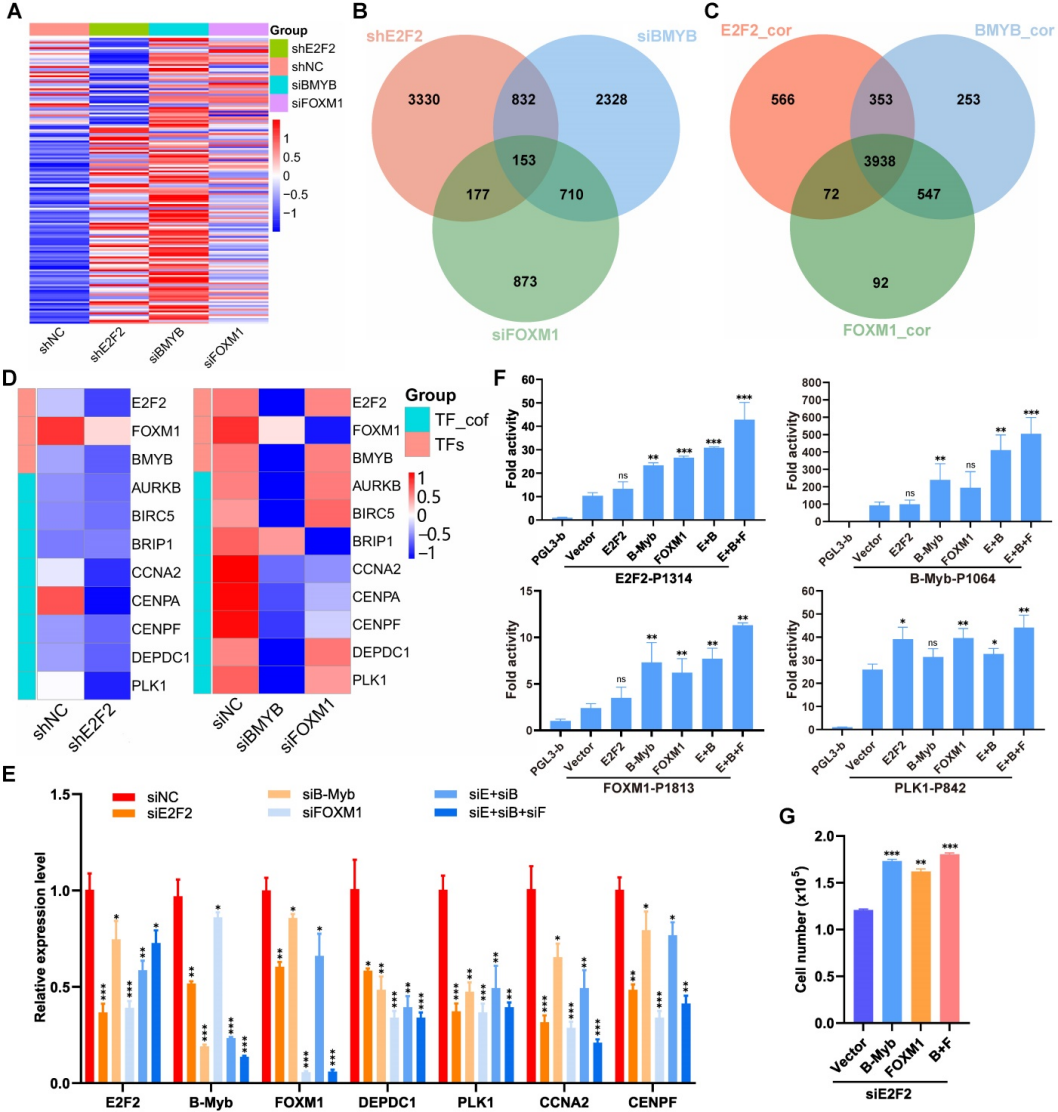
** Interconnected coregulatory circuitry among E2F2, B-Myb and FOXM1.** (**A**) Heatmap of differentially expressed genes after knockdown of E2F2, B-Myb or FOXM1. The RNA-seq data for E2F2 knockdown in A549 cells were obtained in this study as described in Fig. 7A, and the RNA-seq data for B-Myb knockdown and FOXM1 knockdown were obtained from GEO database (GSE143145). (**B**) Venn diagram of differentially expressed genes following silencing of E2F2, B-Myb or FOXM1 as shown in (A). (**C**) Venn diagram of highly correlated genes with E2F2, B-Myb or FOXM1 in TCGA LUAD dataset. E2F2_cor: E2F2_corelated genes; B-Myb_cor: B-Myb_corelated genes; FOXM1_cor: FOXM1_corelated genes. (**D**) Heatmap of the “LUAD module TF network” (Fig. 3A) genes after knockdown of E2F2, B-Myb or FOXM1. TF_cof: TF_cofactors. (**E**) Verification of the “LUAD module TF network” (Fig. 3A) gene expression after knockdown of E2F2, B-Myb, and/or FOXM1 by qRT-PCR. siE+siB: siE2F2 + siB-Myb; siE+siB+siF: siE2F2 + siB-Myb + siFOXM1. (**F**) Luciferase Reporter Assays. The indicated plasmids were transiently transfected into H1299 cells, and then the activities of firefly luciferase were detected according to the description of the “Materials and Methods” section. Data are expressed as fold change normalized to the luciferase activities of cells transfected with the promoter-less control vector (pGL3-basic). E+B: E2F2 + B-Myb; E+B+F: E2F2 + B-Myb + FOXM1. (G) Overexpression of B-Myb and/or FOXM1 rescues the attenuated cell growth phenotype caused by E2F2 knockdown. A549 cells were first transiently transfected with E2F2 siRNA for 24 hours and then transiently transfected with the indicated overexpression plasmids of B-Myb and/or FOXM1 for another 24 hours. Cells were then counted and re-seeded on 24-well plates, and forty-eight hours later cell numbers were counted for cell growth assays. Statistically significant differences between the control group (siNC group in E, Vector group in F and G) and any other experimental group were evaluated using paired t test. P<0.05 (*), P<0.01 (**), P<0.001 (***), nonsignificant (ns).

The “LUAD module TF network” we obtained (Fig. 3A) strongly suggests a specific core regulatory network driven by E2F2, B-Myb and FOXM1 in LUAD. To identify this core regulatory network, we first analyzed the transcriptomic profiles after knockdown of each of the three transcription factors in A549 cells. The gene expression profiling after knockdown of E2F2, B-Myb or FOXM1 showed similarly altered profiling patterns, suggesting that E2F2, B-Myb and FOXM1 cooperatively regulated similar downstream target gene cascades (Fig. 8A and 8B). Consistently, gene expression correlation analysis also indicated that the correlated genes with E2F2, B-Myb or FOXM1 were highly overlapped (Fig. 8C). The RNA-seq data also suggests the mutual regulation among E2F2, B-Myb and FOXM1 (Fig. 8D). qRT-PCR analyses further verified that knockdown of each transcription factor reduced the expression of the other two, suggesting that they regulated each other (Fig. 8E). Knockdown of each or all of the three transcription factors also downregulated the expression of the transcription cofactors in the network such as CCNA2, DEPDC1 and PLK1, which also serve as target genes of E2F2, B-Myb and FOXM1 (Fig. 8E). Furthermore, luciferase reporter assays revealed that, whereas overexpression of each of E2F2, B-Myb and FOXM1 could enhance all their promoter activities as well as that of their downstream target gene PLK1 to some extent, overexpression of E2F2, B-Myb and FOXM1 together could remarkably transactivate all the promoter activities tested, highly suggesting the cooperation among the three transcription factors (Fig. 8F). Functional analysis revealed that overexpression of B-Myb and/or FOXM1 could significantly rescue the attenuated cell growth caused by siRNA-mediated E2F2 knockdown (Fig. 8G).

**Figure 9:**
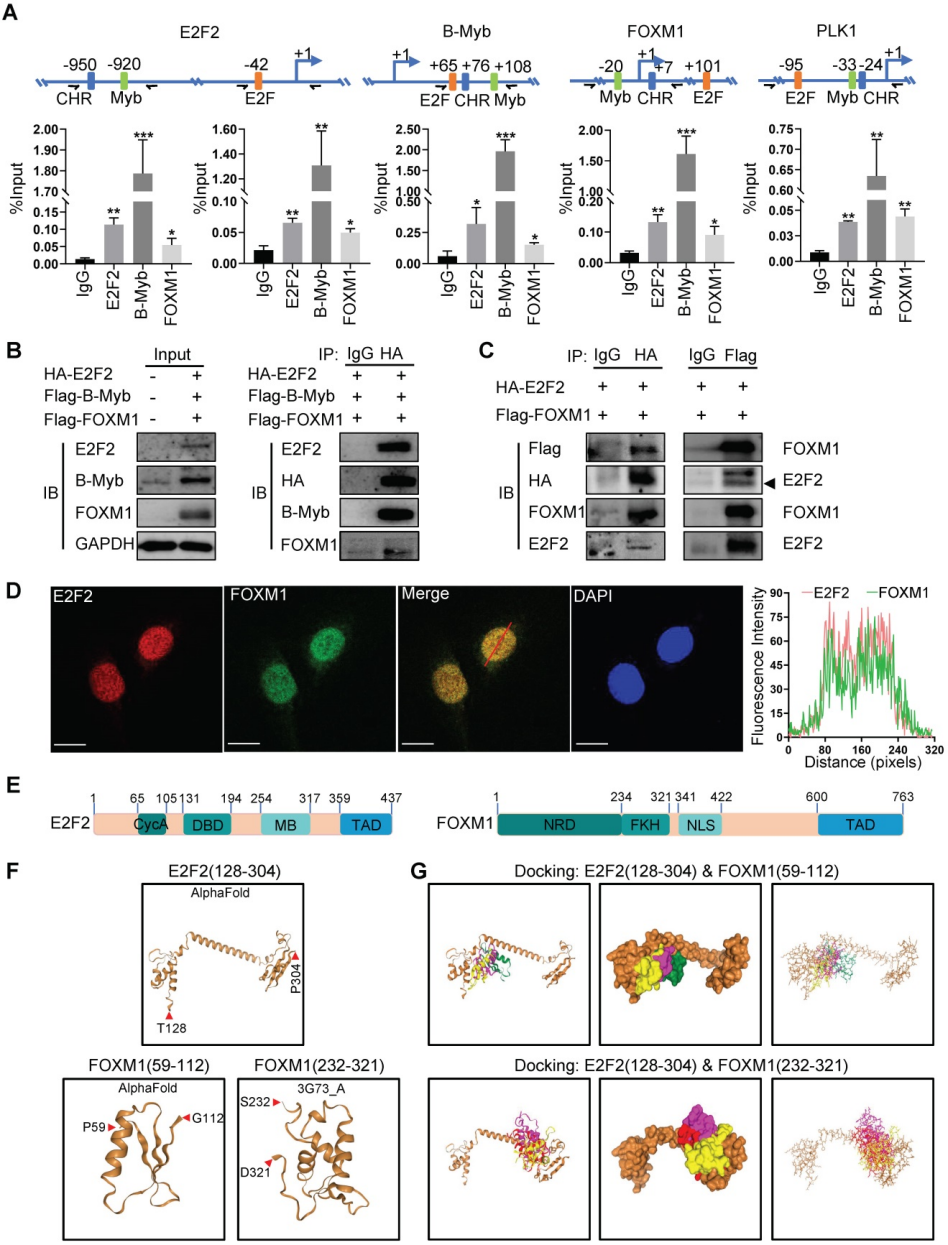
** E2F2, B-Myb and FOXM1 bind to each other's promoter regions *in vivo*.** (**A**) E2F2, B-Myb and FOXM1 bind to the promoter regions of E2F2, B-Myb and FOXM1 as well as their target gene PLK1 *in vivo*. The upper panel shows the schematic diagram of E2F and Myb binding sites and cell cycle homology element (CHR, bound by FOXM1) in the corresponding gene promoter regions. The transcription start sites are indicated as +1. Paired arrows indicate the upstream and downstream primers for ChIP qPCR. Lower panels are the calculated data of ChIP qPCR assays performed in A549 cells with the indicated specific antibodies and control IgG. The data are expressed as percent of recovered immunoprecipitated DNA with respect to the Input DNA. (**B**) E2F2, B-Myb and FOXM1 associate with each other *in vivo*. H1299 cells were transiently transfected with the indicated plasmids for 48hs, and then cell lysates were prepared and subjected to co-immunoprecipitation assays. (**C**) E2F2 associates with FOXM1 *in vivo*. Co-immunoprecipitation assays were performed as in (B). (**D**) E2F2 colocalizes with FOXM1 in cell nuclei. H1299 cells were transfected with pCDH-puro-HA-E2F2 and GV365-3×Flag-FOXM1b expression plasmids for 48h, and then cells were fixed and stained with anti-E2F2 antibody (red) and anti-FOXM1 antibody (green), and intensity spatial profiles were plotted. Scale bar = 10 µm. (**E**) Schematic illustration of the primary structures of E2F2 and FOXM1. Cyc A, Cyclin A/CDK2 binding domain; DBD, DNA-binding domain; MB, marked box; TAD, Transactivation domain; NRD, N-terminal Repressor Domain; FKH, Forkhead DNA Binding domain; NLS, Nuclear Localization Signal. (**F**) Three-dimensional (3D) structures of E2F2 and FOXM1. The 3D structures of E2F2(128-304) and FOXM1(59-112) were downloaded from AlphaFold, and the 3D structure of FOXM1(232-321) was obtained from RSCB PDB and HDOCK. (**G**) Predicted models of E2F2-FOXM1 protein docking. The top three homologous docking models for E2F2 and FOXM1 interaction were predicted by HDOCK server online and presented with three differentially colored 3D structures of FOXM1.

Transcription factor binding analysis revealed that the promoters of E2F2, MYBL2 and FOXM1 as well as that of PLK1 all contains potential biding sites for E2F2, MYBL2 and FOXM1 (Fig. 9A). ChIP assays showed that E2F2, B-Myb and FOXM1 bind to the promoter regions of each other as well as that of their cofactor target gene PLK1 *in vivo* (Fig. 9A). Co-immunoprecipitation assays revealed that E2F2, B-MYB and FOXM1 associated with each other in a complex (Fig. 9B). As the interactions of E2F2 and B-Myb, and B-Myb and FOXM1 have been reported by our group and others 21, 37, 38, we then further investigated the potential association between E2F2 and FOXM1. Co-immunoprecipitation assays with either E2F2 or FOXM1-tagged antibodies confirmed that E2F2 associated with FOXM1 *in vivo* (Fig. 9C). Immunofluorescence staining showed that E2F2 co-localized with FOXM1 in cell nuclei (Fig. 9D). Computational docking analysis of E2F2 and FOXM1 through the HDOCK server revealed significant top-scored homologous docking models of interaction between N-terminal region of E2F2 and N-terminal region or middle region of FOXM1 (Fig. 9E, 9F, 9G). Taken together, these results suggest that E2F2, B-Myb and FOXM1 associate with each other and transcriptionally regulate the expression of each other as well as their target genes, thus forming an interconnected coregulatory circuitry in LUAD.

### Therapeutic and prognostic values of E2F2-transactivated regulatory circuitry

We and others have previously demonstrated the *in vivo* effects of B-Myb and FOXM1 on LUAD 29, 39, 40, whereas the role of E2F2 remains unclear. As shown in Fig. 9A and 9B, compared with the control group, overexpression of E2F2 significantly increased the tumor growth in nude mice, while knockdown of E2F2 completely abolished tumorigenesis, indicating that E2F2 is essential for and can promote tumor growth *in vivo* in LUAD.

Furthermore, we evaluated the prognostic values of the 11 genes of “LUAD module TF network” in LUAD patients through univariate Cox regression analysis. The results revealed that totally 10 genes including B-Myb, FOXM1, PLK1, DEPDC1, CCNA2, CENPF, CENPA, AURKB, BRIP1 and BRIC5, were significantly correlated with overall survival of LUAD patients (Table S6). The prognostic value of 11 genes was also examined by Kaplan-Meier curve, the p-values were calculated using log-rank test (Fig. 9C and Fig. S4). Next, the stepwise regression method of multivariate Cox analysis is used to establish the optimal regression subset. In univariate regression analysis, it is often impossible to identify whether confounding factors exist, and confounding factors are likely to interfere with the relationship between variables and outcomes. It is unreasonable to include only statistically significant variables in univariate analysis into multivariate COX analysis. Therefore, all 11 genes were included in multivariate Cox regression analysis. Two prognostic genes (E2F2, PLK1) were finally included in the model for calculating the Risk score. The coefficients were provided in Table S6. The risk score of patients were calculated using the following formula:

Risk score = (1.624 * exp(PLK1)) + (0.712 * exp(E2F2)) (3)

The Kaplan-Meier curve was employed to test the prognostic value of the model (Fig. 9D); patients with a high-risk score exhibit the significantly poorer survival prognosis compared with the low-risk group. Experimental verification results and bioinformatics analysis results indicate that B-Myb is likely to be a prognostic target. In the multivariate cox regression results, the T-test P value of B-Myb is 0.054, so we also included B-Myb in the model and constructed a second prognostic model. The KaplanMeier curve is shown in Fig. 9D, and the model coefficients are in Table S7.

## Discussion

### E2F2 is an essential tumor-promoting gene in LUAD

E2F2 is a canonical member of the E2F family of transcription factors including three distinct sub-categories, i.e, activators of E2F1-3, canonical repressors of E2F4-5, and atypical repressors of E2F7-8 10, 11. Like E2F1 and E2F3, E2F2 has been well demonstrated to play a central role in regulating cell cycle progression, proliferation and oncogene-mediated transformation 13, 41, 42. E2F1 and E2F3 have been shown to be overexpressed and exert tumor-promoting effects in several types of cancers including lung cancer 10, 17, 43-45. However, the study on the function of E2F2 in tumorigenesis is quite limited. E2F2 has been reported to be overexpressed in several cancers such as ovarian cancer, breast cancer, colorectal cancer, etc. 10, 17, 18. It is noteworthy that high incidence of thymic epithelial tumors was observed in E2F2 transgenic mice 19. Reimer et al demonstrated that among eight distinct E2F family members, especially E2F2 plays a pivotal role in tumorigenesis of ovarian cancer 17, 18. Consistently, our recent study demonstrated that compared with E2F1 and E2F3, E2F2 might specifically play a pivotal role in colorectal cancer and serve as a specific therapeutic target.

However, although two studies by bioinformatic analysis revealed that E2F2 mRNA is overexpressed and has prognostic value in The Cancer Genome Atlas (TCGA) and Gene Expression Omnibus (GEO) lung cancer datasets 46, 47, the function of E2F2 in LUAD remains largely elusive. In the present study, we found that the expression of E2F2 was significantly elevated in LUAD. Our functional analysis revealed that E2F2 overexpression remarkably promotes LUAD cell cycle progression, proliferation and motility, whereas E2F2 knockdown represses the malignant phenotypes. Subsequent *in vivo* xenograft nude mouse models further verified the *in vitro* growth-promoting effects. All the results together highly suggest that E2F2 essentially contributes to LUAD progression by promoting cell cycle progression, cell proliferation and motility. Our present findings are in accordance with previous observations describing the oncogenic role of E2F2 in other cancers.

### Exquisite transcription regulatory circuitry of E2F2, B-Myb and FOXM1 in LUAD

To date, E2F2 as well as its activator counterparts of E2F1 and E2F3 has been demonstrated to exert functions mainly via two mechanisms. Firstly, E2F2 directly binds to the target gene promoters and transactivates the expression of downstream target genes that regulate cell cycle progression, apoptosis, and metastasis 9, 11. Secondly, E2F2 can directly interact with other proteins to execute its functions. In addition to its well-known binding partners of the transcription factor dimerization partner (TFDP) family members (TFDP1-3) and the pocket proteins (RB, p107 and p130), previous studies have also demonstrated that E2F2 could interact with RING1 and YY1 binding protein (RYBP), nuclear protein Aly/REF export factor (ALYREF, also known as THOC4 or ALY), CREB and γ-tubulin 48-51.

Recently, we have demonstrated that E2F2 associate with B-Myb, and more intriguingly both transcription factors mutually regulate each other, thus forming an exquisite reciprocal feed-forward loop which plays a vital role in accelerating colorectal cancer progression 21. Feed-forward loop regulation is a common motif of transcriptional regulatory networks in prokaryotes and metazoans, and represents an effective strategy of transcriptional control program to stabilize and enforce cell phenotype 52-54. In this study, our results again revealed that the E2F2/B-Myb feed-forward loop also exists in LUAD, reinforcing its biological significance in general. Moreover, we further revealed that E2F2 and B-Myb, along with FOXM1, mutually regulate each other's expression, associate with each other, and thus constitute a consolidated core transcription regulatory circuitry that contributes to the malignant progression of human LUAD. As a widespread network motif, the core transcription regulatory circuitry plays a fundamental role in establishment and maintenance of cell identity 54, 55. Given the multiple roles of E2F2, B-Myb and FOXM1 in various biological processes including cell growth, cell cycle, invasion, apoptosis and cell senescence, and several types of cancers including colorectal cancer and LUAD 21, 40, 56-61, the core transcription regulatory circuitry of E2F2/B-Myb/FOXM1 would be of broad physiological and pathological significances which warrants deep investigations in future.

It is important to note that the promoter regions of E2F2, B-Myb and FOXM1 as well as their target genes such as PLK1 all contain binding sites for E2F2, B-Myb and FOXM1. ChIP assays revealed the *in vivo* binding of E2F2, B-Myb and FOXM1 to these elements. Co-IP assays demonstrated the molecular association among these three transcription factors. Thus, the data suggest that E2F2, B-Myb and FOXM1 might work as a complex to regulate each other as well as their target genes. E2F2 and B-Myb regulate target gene transcription through binding to the consensus E2F-binding sites and Myb-binding sites and/or interacting with other transcription factors/cofactors 9-11, 21, 57. Of note, FOXM1 could control gene expression of cell cycle related genes mainly through being recruited to CHR (cell cycle genes homology region) elements via protein-protein interaction, but not to the canonical forkhead binding motifs 38, 62. Numerous studies demonstrated that transcription activation requires trans-recruitment of transcription factor complex to the regulatory regions of target genes 63, 64. Our lab is currently investigating the detailed functional implication of E2F2, B-Myb and FOXM1-containing complex in transcriptional regulation and cancer development, which is of great scientific importance and biological significance.

### Diagnostic and therapeutic values of PLK1 against E2F2-mediated network in LUAD

Our results highly suggest that the E2F2-centered transcriptional regulatory network plays a pivotal role in LUAD progression and might serve as a promising diagnostic and therapeutic target for the treatment of LUAD. However, as a transcription factor, E2F2 as well as B-Myb and FOXM1 is currently undruggable. Our prognostic analysis on the E2F2-centered network genes revealed that a E2F2/PLK1 two-gene signature remarkably stratified LUAD patients into low- and high-risk groups, suggesting that PLK1 might act as a pivotal regulator in the E2F2-centered network.

Previous reports showed that E2F1 could directly regulate the expression of PLK1 65, 66. Our present study further verified that PLK1 is direct transcriptional target of E2F2. Previous studies also reported that FOXM1 directly regulates the expression of PLK1 through an atypical chromatin binding mechanism, and PLK1 further phosphorylates and hyperactivates FOXM1, thus forming a positive feedback loop 10, 16, 67-69. B-Myb has been also demonstrated to regulate the expression of PLK1 via direct binding to PLK1 gene promoter 37, 70. Werwein et al reported that sequential phosphorylations of B-Myb by PIN1, CDK2 and PLK1 are essential for B-Myb to transactivate its mitotic target genes 61. Taken together, PLK1 might act as not only a critical downstream target but also a pivotal upstream regulator for the E2F2-centered network in LUAD. PLK1 is an evolutionary conserved serine/threonine kinase, and plays important roles in several biological processes such as cell cycle, autophagy and apoptosis 71-73. Numerous studies have demonstrated that PLK1 is overexpressed in many cancers correlating with poor prognosis, making PLK1 as a promising target for cancer treatment. Several specific small molecular PLK1 inhibitors have been developed and entered phase I and II clinical studies for patients with various cancers 73. Therefore, further studies are needed to investigate the therapeutic values of PLK1 inhibitors against E2F2-mediated network in LUAD.

## Conclusion

In summary, our results clarified that E2F2 essentially contributes to LUAD progression by promoting cell cycle progression, cell proliferation and motility, and further unraveled an exquisite core transcription regulatory circuitry of E2F2/B-Myb/FOXM1 that contributes to the malignant progression of human LUAD.

## Supplementary Material

Supplementary figures and tables.

## Figures and Tables

**Figure 10:**
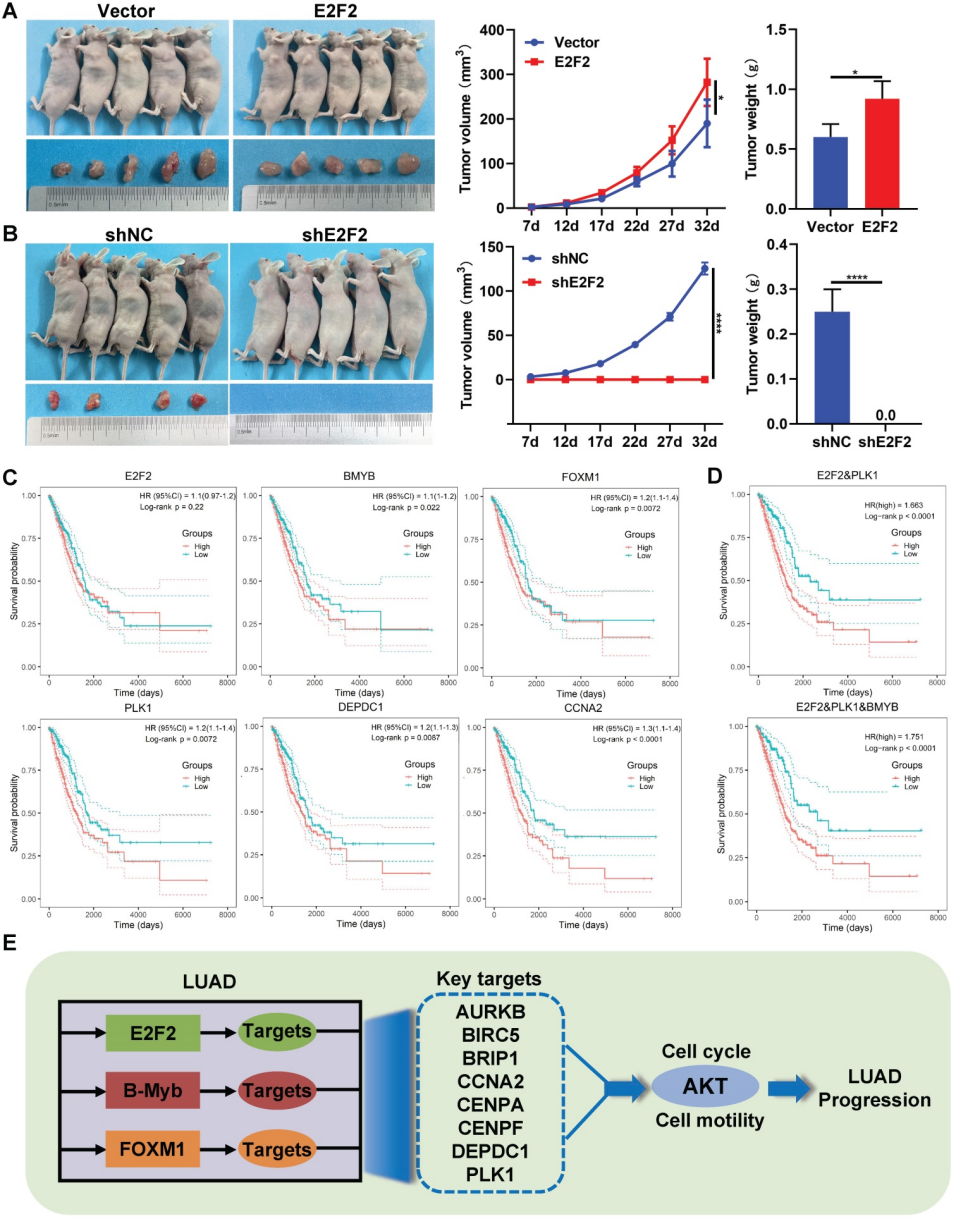
** Therapeutic and prognostic values of E2F2-transactivated circuitry.** (**A-B**) E2F2 promotes LUAD growth *in vivo*. E2F2 overexpression and knockdown A549 cells were injected subcutaneously into the dorsal flanks of nude mice. The tumors were monitored regularly for 5 weeks and excised at the end of the experiment. (**C**) Relationship between overall survival and expression levels of the seven “LUAD module TF network” genes. Probabilities for overall survival were estimated using the Kaplan-Meier method. (**D**) Multiple gene prognostic signature performance in LUAD patients. Kaplan-Meier curves of overall survival were stratified by multiple gene prognostic signature in high and low risk. (**E**) Schematic model of E2F2/B-Myb/FOXM1 core regulatory circuitry in LUAD.
