# Supplementary material for: E2F2 promotes lung adenocarcinoma progression through B-Myb- and FOXM1-facilitated core transcription regulatory circuitry
Source: Int J Biol Sci. 2022 Jun 25;18(10):4151–70. doi: 10.7150/ijbs.72386 (PMC9274503; doi:10.7150/ijbs.72386)
Supplement: Supplementary file 1 — Supplementary figures and tables. [file ijbsv18p4151s1.pdf]

Supplementary Figures

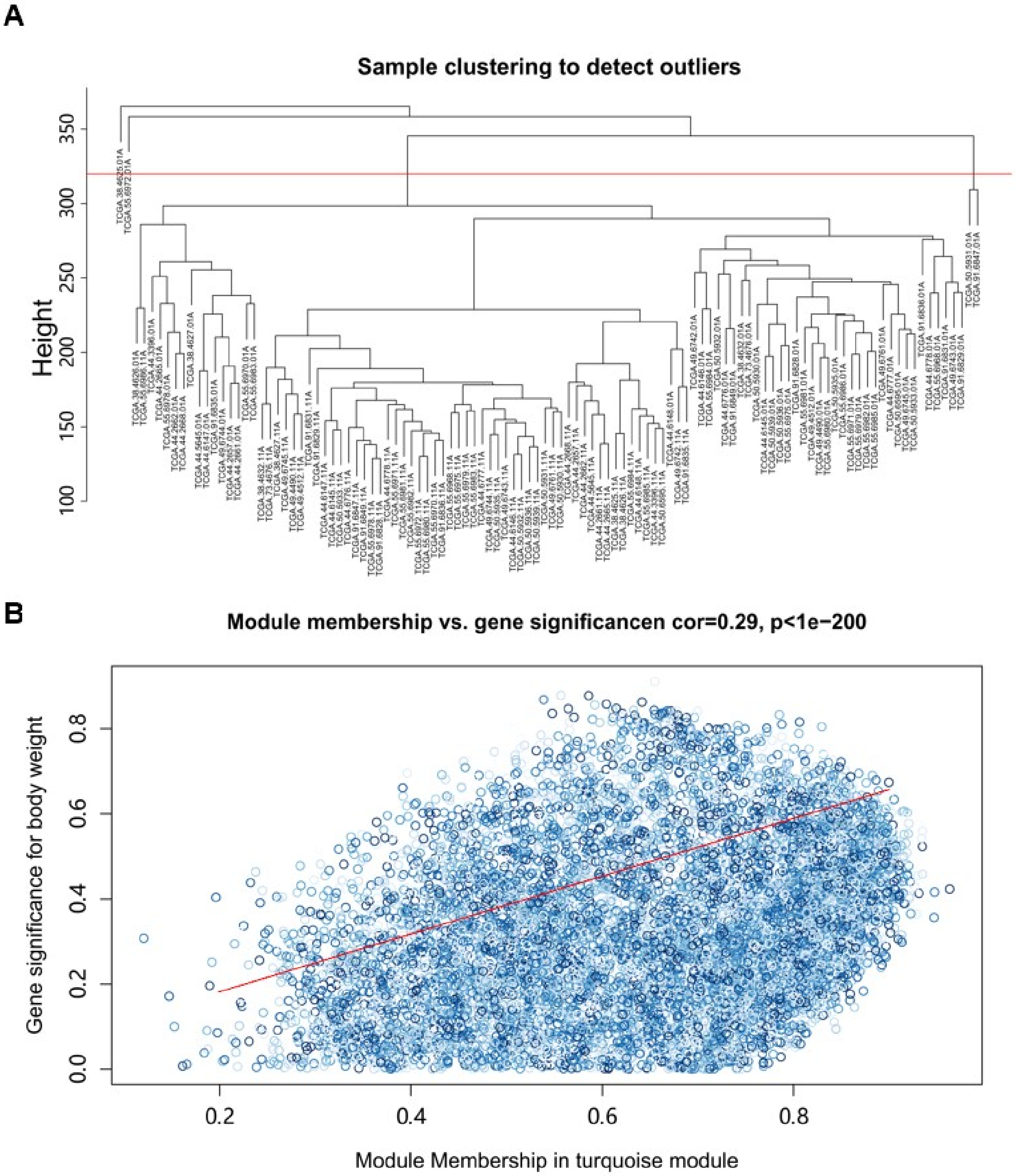

Figure S1 Sample clustering and module membership.

(**A**) Cluster analysis of samples to detect outliers. The four outlier samples above the red line were removed. (**B**) The correlation coefficient between turquoise module membership and gene significance.

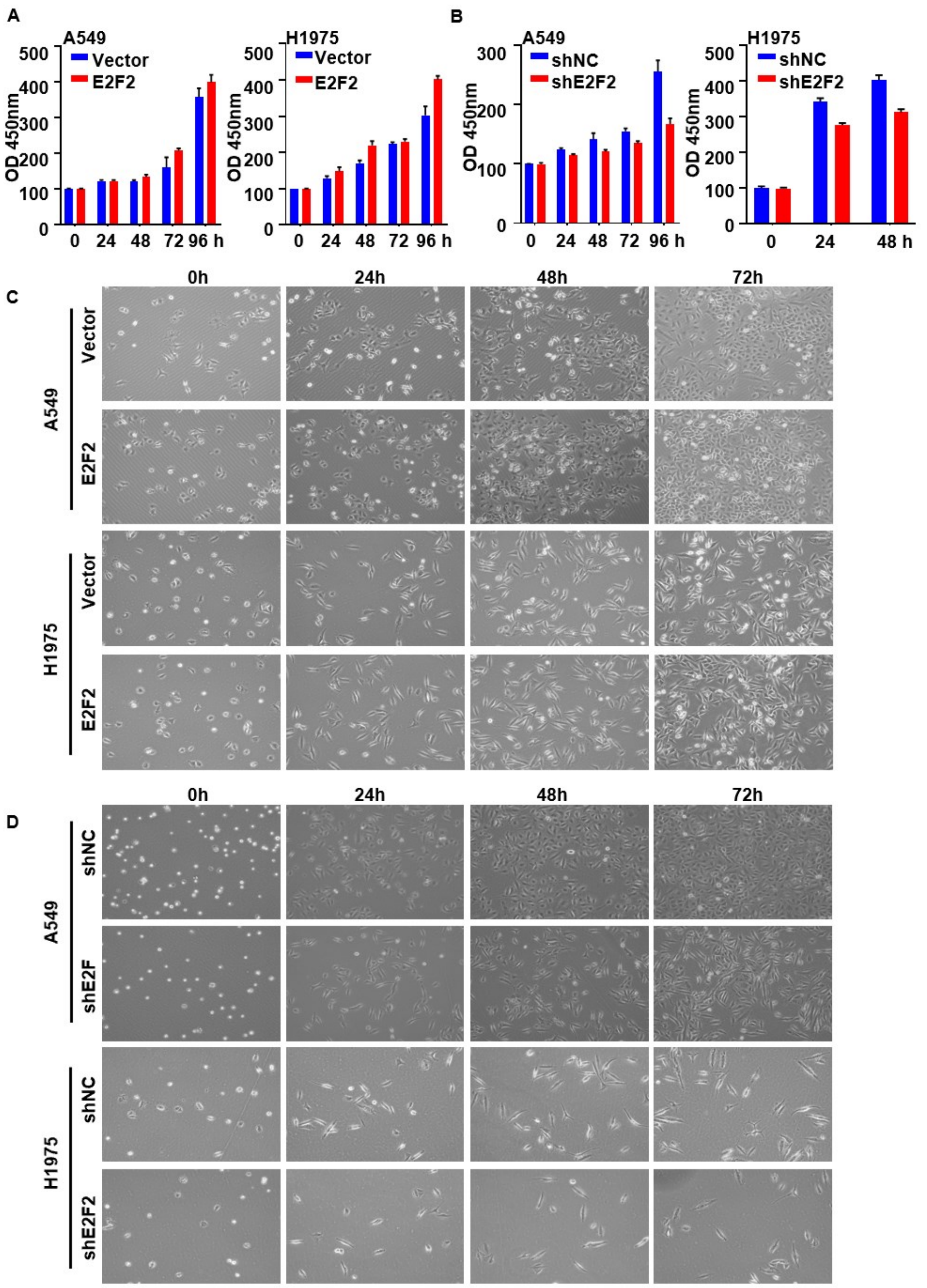

Figure S2 E2F2 accelerates LUAD cell growth.

The stable E2F2 knockdown or overexpression cells were used for cell proliferation assays and cell morphology. (**A-B**) Cell proliferation assays. CCK8 assays were used to detect cell proliferation at the indicated time-points. (**C-D**) Cell morphology. Cell morphology was observed by phase-contrasted microscope at the indicated time-points (×100).

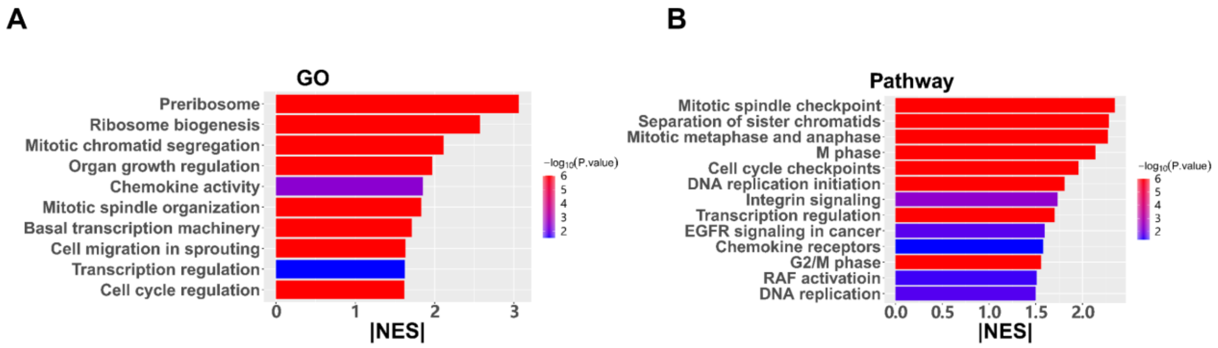

Figure S3 GSEA analysis on gene expression profiling after E2F2 knockdown.

(**A-B**) GO enrichment analysis. (**B**) Pathway enrichment analysis. Normalized enrichment score, NES.

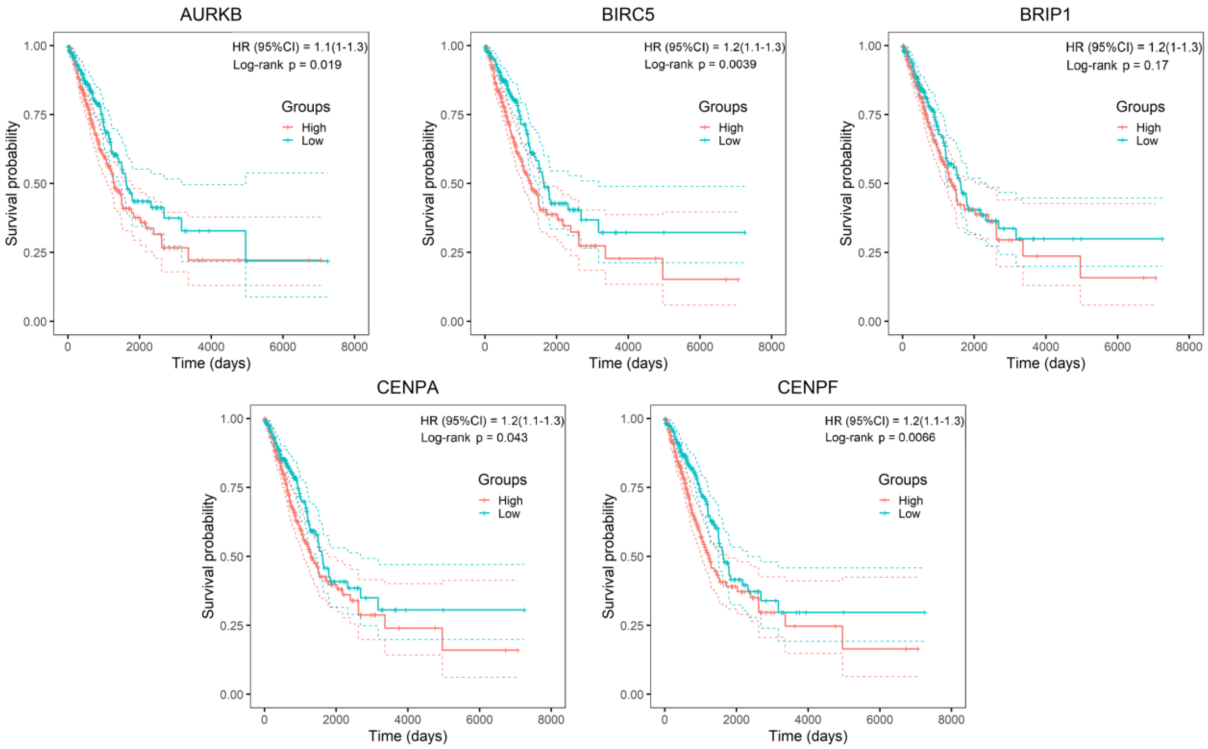

Figure S4 Overall survival of LUAD module TF network genes.

(**A-D**) Relationship between overall survival and expression levels of AURKB, BIRC5, BRIP1 and CENPF genes in LUAD module TF network.

Supplementary Tables

Table S1. Patient baseline characteristics.

Characteristic	LUAD patients (n = 55)
**Age, year (mean ± SD)**	66.3±10.8
**Male, n (%)**	23(41.8)
**Smoking, PY (mean ± SD)**	39.9±22.2
**TNM stage, n (%)**	
not reported	1(1.8)
I	26(47.3)
II	13(23.6)
III	13(23.6)
IV	2(3.6)
**Deaths (%)**	46.43

Table S2. Primers, shRNAs, siRNAs and plasmids used in this study.
Primers used for qRT-PCR analyses

Gene name	Primer name and sequences
GAPDH	F833: ACCTGACCTGCCGTCTAGAA<br>R1060: TCCACCACCCTGTTGCTGTA
E2F1	F337: TGGACTTCACCTTGGGAGAC<br>R535: TCAGTCACATCAGGGCTTGT
E2F2	F1370: AGCAGAGTTTTGGGATGGGA<br>R1599: CAGACAGGACACGAAATGCC
E2F3	F1110: GGAAGTGAAAACATTGGCCG<br>R1299: ATGTTCTTGTACGCCCTCCA
B-Myb	F484: AAGATGTTGCCAGGGAGGAC<br>R679: TGGTCAGAAGACTTCCCTGG
FOXM1	F1071: ATACGTGGATTGAGGACCACT<br>R1245: TCCAATGTCAAGTAGCGGTTG
TOP2A	F12: ACCATTGCAGCCTGTAAATGA<br>R140: GGGCGGAGCAAAATATGTTCC
GRB2	F438: ATTCCTGCGGGACATAGAACA<br>R633: GGTGACATAATTGCGGGGAAAC
HMGA1	F114: CGAAGTGCCAACACCTAAGAG<br>R222: GCCCCTTGGTTTCCTTCCT
NOB1	F61: CAGGACATCGGGAAGAACATTT<br>R192: CCGCACGTATTCCGGTAAGG
MYBBP1A	F219: CCTGAAGCGTCTAATCACGGG<br>R429: GAGGGCGAGCACTCCAAAC
BOP1	F643: GTGGGCTTCAACCCCTATGAG<br>R778: CCATGCGAGAGACCTTCTCC
PLK1	F328: CACCAGCACGTCGTAGGATTC<br>R474: CCGTAGGTAGTATCGGGCCTC
CCNA2	F444: GGATGGTAGTTTTGAGTCACCAC<br>R645: CACGAGGATAGCTCTCATACTGT
CDC20	F4：GCACAGTTCGCGTTCGAGA<br>R191：CTGGATTTGCCAGGAGTTCGG
CCND1	F461：CAATGACCCCGCACGATTTC<br>R606：CATGGAGGGCGGATTGGAA
CENPF	F1198：CTCTCCCGTCAACAGCGTTC<br>R1299：GTTGTGCATATTCTTGGCTTGC

Primers used for ChIP-PCR analyses

Gene name	Primer name and sequences
E2F2	E2F2-ChIP-F1113： AATAATACAACTGTGAACAAGACAAAGC<br>E2F2-ChIP -R1285: CATACAGAGAGATTCCCTGATTTGA
E2F2	E2F2-ChIP -F1930： CGGGAGATCCGAATTTC<br>E2F2-ChIP -R2037： CCTTGCGGCTCGCTCTCTA
B-Myb	BMYB-ChIP -F 2063： AGATAGAAAAGTGCTTCAACCCG<br>BMYB -ChIP-R 2153： CTCGAAGGCGTCAGCGTGT
FOXM1	FOXM1-ChIP-F 1908: GACCGCACAGCCTTCGAG<br>FOXM1-ChIP-R 2034: CACCGGAGCTTTCAGTTTGT
PLK1	PLK1-ChIP-F1824: CGTCCGTGTCAATCAGGTTTTC<br>PLK1-ChIP-R2003: CTCCGCTCCTCCCCGAAT

Primers for luciferase reporter constructions

Constructs	Methods	Primers and/or Enzymes used
FOXM1-P1813	PCR based cloning	PF519: GCTAGCCCGGGCTCGAGATCTATCCAACTGTTCTGCCCTAA *Bgl II*
		PR2331: GTACCGGAATGCCAAGCTTCCTGCTGCTAGACGCCCTGA *HindIII*
PLK1-P842	PCR based cloning	PF1028: ACCGAGCTC CGGTGGCTCACGCCTGTAATCC Sac I
		PR2049: CTAGCTAGC CCGAAGCTGCGCTGCAGACCTC Nhe I

shRNA sequences.

Gene name	Primer name and sequences
Negative control shRNA (NCsh)	Sense: 5'-*ACCGGT*TGGTTTACATGTTGTGTGACTCGAG TCACACAACATGTAAACCATTTTT-3'<br>Antisense: 5'- *GAATTC*AAAAATGGTTTACATGTTGTGTGACTCGAG TCACACAACATGTAAACCA-3'
E2F2 shRNA (E2F2sh)	Sense: 5'-*ACCGGT* GCCTATGTGACTTACCAGGATCTCGAGATCCTGGTAAGTCACATAGGCTTTTT-3'<br>Antisense: 5'- *GAATTC*AAAAAGCCTATGTGACTTACCAGGATCTCGAG TCACACAACATGTAAACCA -3'

Plasmids

Constructs	Application	Sources
pCDH-puro-HA-E2F2	Expression vectors	Our lab
LV105-Flag-B-Myb	Expression vectors	Our lab
GV365-3×Flag-FOXM1c	Expression vectors	Our lab

E2F2-P1314(-984/+329)	Promoter reporter	Fan et al 2021
BMYB-P1064(-916/+148)	Promoter reporter	Fan et al 2021
PLK1-P842	Promoter reporter	Our lab
FOXM1-P1813(-1482/+331)	Promoter reporter	Our lab
PLP1	packaging plasmid	Cui lab
PLP2	packaging plasmid	Cui lab
PLP/VSVG	packaging plasmid	Cui lab
pMD2.G	packaging plasmid	Yu lab
psPAX2	packaging plasmid	Yu lab

Table S3. Antibodies used in the present study.

Protein name	Manufacture (cat. number)	Applications (working dilution)	Website Link
GAPDH	Xianzhi Bio (AB-P-R 001)	IB (1:5000)	http://www.goodhere.com/showproduct.asp?id=320&classid=34&nid=2
B-Myb	Santa Cruz (N-19):sc-724	IB (1:500)<br>ChIP	http://www.scbt.com/datasheet-724-b-myb-n-19-antibody.html
E2F2	Santa Cruz (TFE-25):sc-9967	IB (1:500)<br>IF (1:200)<br>ChIP	https://www.scbt.com/scbt/zh/product/e2f-2-antibody-tfe-25?requestFrom=search
FOXM1	Cell Signaling Technology (D3F2B, 20459)	ChIP	https://www.cellsignal.cn/products/primary-antibodies/foxm1-d3f2b-rabbit-mab/20459
AKT	Cell Signaling Technology(9272)	IB (1:1000)	https://www.cellsignal.cn/products/primary-antibodies/akt-antibody/9272
p-Akt	Cell Signaling Technology (4060)	IB (1:500)	http://rlgene.com/showproduct.asp?/1_439
HA	Beyotime (AH158)	IF (1:300)	http://www.beyotime.com/product/AH158.htm
pHH3	Cell Signaling Technology (9706)	IF (1:200)	https://www.cellsignal.com/products/primary-antibodies/phospho-histone-h3-ser10-6g3-mouse-mab/9706?N=4294956287&Ntt=Phospho+Histone+H3++ser10&fromPage=plp
IgG	Beyotime (A7016)	IP	https://www.beyotime.com/product/A7016.htm
Flag	Sigma (F1804-200UG)	IP	https://www.sigmaaldrich.com/catalog/product/sigma/f1804?lang=zh&region=CN
antiMouse secondary antibody	Abgent (ASS1007)	IB (1:5000)	http://www.abgent.com/products/ASS1007-Goat-Anti-Mouse-IgGHL-Human-ads-HRP-Secondary-Antibody
antiRabbit secondary antibody	Abgent (ASS1009)	IB (1:5000)	http://www.abgent.com/products/ASS1009-Goat-Anti-Rabbit-IgGHL-MouseHuman-ads-HRP-Secondary-Antibody

antiMouse seccondary antibody	Thermo (A-11031)	IF (1:1000)	https://www.thermofisher.com/antibody/product/Goat-anti-Mouse-IgG-H-L-Secondary-Antibody-Polyclonal/A-11031
antiRabbit seccondary antibody	Thermo (A-11034)	IF (1:1000)	https://www.thermofisher.com/antibody/product/Goat-anti-Rabbit-IgG-H-L-Secondary-Antibody-Polyclonal/A-11034

Table S4. GO and Pathway analysis of dysregulated genes in E2F2 knockdown cells.
Top list of GO enrichment analysis.

Term ID	Description	P value	Count
GO:0006260	DNA replication	7.81E-10	27
GO:0010469	Regulation of signaling receptor activity	4.33E-09	43
GO:0007155	Cell adhesion	2.09E-07	57
GO:0000082	G1/S transition of mitotic cell cycle	3.56E-07	18
GO:0007010	Cytoskeleton organization	1.22E-05	22
GO:0051315	Attachment of mitotic spindle microtubules to kinetochore	2.16E-05	5
GO:0042060	Wound healing	5.50E-05	16
GO:0070374	Positive regulation of ERK1 and ERK2 cascade	1.66E-04	25
GO:0008283	Cell proliferation	2.53E-04	38
GO:0030198	Extracellular matrix organization	3.57E-04	22

Top list of Pathway enrichment analysis.

Term ID	Description	P value	Count
4060	Cytokine-cytokine receptor interaction	1.82E-11	42
3030	DNA replication	7.55E-07	11
4110	Cell cycle	4.68E-06	20
4010	MAPK signaling pathway	3.23E-05	32
4064	NF-KB signaling pathway	5.24E-05	16
4668	TNF signaling pathway	4.96E-05	18
4350	TGF-beta signaling pathway	1.21E-04	13
5200	Pathways in cancer	0.00116	42
4151	PI3K-Akt signaling pathway	0.00604	30
4014	Ras signaling pathway	0.00716	20

Table S5. GSEA analysis of gene expression profile after E2F2 knockdown.

Top list of enriched GOs.

Term ID	Description	P value	Count
GO:0006260	DNA replication	7.81E-10	27
GO:0010469	Regulation of signaling receptor activity	4.33E-09	43
GO:0007155	Cell adhesion	2.09E-07	57
GO:0000082	G1/S transition of mitotic cell cycle	3.56E-07	18
GO:0007010	Cytoskeleton organization	1.22E-05	22
GO:0051315	Attachment of mitotic spindle microtubules to kinetochore	2.16E-05	5
GO:0042060	Wound healing	5.50E-05	16
GO:0070374	Positive regulation of ERK1 and ERK2 cascade	1.66E-04	25
GO:0008283	Cell proliferation	2.53E-04	38
GO:0030198	Extracellular matrix organization	3.57E-04	22

Top list of enriched Pathways.

Term ID	Description	P value	Count
4060	Cytokine-cytokine receptor interaction	1.82E-11	42
3030	DNA replication	7.55E-07	11
4110	Cell cycle	4.68E-06	20
4010	MAPK signaling pathway	3.23E-05	32
4064	NF-KB signaling pathway	5.24E-05	16
4668	TNF signaling pathway	4.96E-05	18
4350	TGF-beta signaling pathway	1.21E-04	13
5200	Pathways in cancer	0.00116	42
4151	PI3K-Akt signaling pathway	0.00604	30
4014	Ras signaling pathway	0.00716	20

Table S6. Univariate and multivariate Cox regression analysis in LUAD cohort.

Variables	Univariate analysis		Multivariate analysis	
	HR (95%CI)	P-value	Coefficients	P-value
AURKB	1.1(1-1.3)	0.0041		0.126
BIRC5	1.2(1.1-1.3)	0.00087		0.338
BRIP1	1.2(1-1.3)	0.007		0.880
CCNA2	1.3(1.1-1.4)	0.000032		0.779
CENPA	1.2(1.1-1.3)	0.0015		0.235
CENPF	1.2(1.1-1.3)	0.00049		0.329
DEPDC1	1.2(1.1-1.3)	0.00027		0.968
E2F2	1.1(0.97-1.2)	0.16	0.712	0.001
FOXM1	1.2(1.1-1.4)	0.00012		0.558
BMYB	1.1(1-1.2)	0.0068		0.054
PLK1	1.3(1.2-1.5)	0.0000072	1.624	0.000

a. Method = Forward Stepwise (Likelihood Ratio)

b. Log-likelihood ratio test $\chi2=32.550, P<0.0001$

Table S7. Regression coefficients of the E2F2/BMYB/PLK1 trio gene prognosis model

Variables	Multivariate analysis	
	Coefficients	P-value
E2F2	0.777	0.003
BMYB	0.808	0.054
PLK1	1.974	0.000

a. Method = Forward Stepwise (Likelihood Ratio)

b. Log-likelihood ratio test $\chi 2$=36.242, P<0.0001
